# The synthesis of a Bi_2_MoO_6_/Bi_4_V_2_O_11_ heterojunction photocatalyst with enhanced visible-light-driven photocatalytic activity†

**DOI:** 10.1039/c7ra12766a

**Published:** 2018-01-31

**Authors:** Chol-Nam Ri, Song-Gol Kim, Kyong-Sik Ju, Hyok-Su Ryo, Chol-Ho Mun, U-Hyon Kim

**Affiliations:** Institute for Electronic Materials, Kim Il Sung University Pyongyang Democratic People's Republic of Korea; School of Chemistry and Chemical Engineering, Harbin Institute of Technology Harbin 150001 China rcn906@yahoo.com; Institute of Advanced Science, Kim Il Sung University Pyongyang Democratic People's Republic of Korea; Faculty of Physics, Kim Il Sung University Pyongyang Democratic People's Republic of Korea

## Abstract

A novel Bi_2_MoO_6_/Bi_4_V_2_O_11_ heterostructured photocatalyst was successfully fabricated using a facile one-pot solvothermal method. This heterojunction consists of homogeneous dispersed Bi_4_V_2_O_11_ nanocrystals anchored on the surface of Bi_2_MoO_6_ nanoflakes, endowing the heterojunction with nanosized interfacial contact. Based on the favorable interfacial contact, the band alignment at the heterojunction effectively facilitated photo-generated carrier transfer, which was verified by photoelectrochemical and photoluminescence measurements. Thereby, in contrast with pristine Bi_2_MoO_6_ and Bi_4_V_2_O_11_, the as-synthesized heterojunction with nanoscale contact exhibited significantly enhanced photocatalytic activity towards the degradation of MB and the reduction of Cr(vi). In addition, the as-fabricated Bi_2_MoO_6_/Bi_4_V_2_O_11_ heterojunction exhibited good cycling stability for MB degradation after 4 cycles. Finally, a plausible photocatalytic mechanism for MB degradation over the Bi_2_MoO_6_/Bi_4_V_2_O_11_ heterojunction was discussed in detail. This work not only reports a highly efficient photocatalyst but also sheds light on the design and optimization of a heterojunction.

## Abstract 2

A Bi_2_MoO_6_/Bi_4_V_2_O_11_ heterojunction exhibits remarkably enhanced photocatalytic activity for MB degradation and Cr(vi) reduction under visible-light illumination compared to its pristine samples.

## Graphical abstract

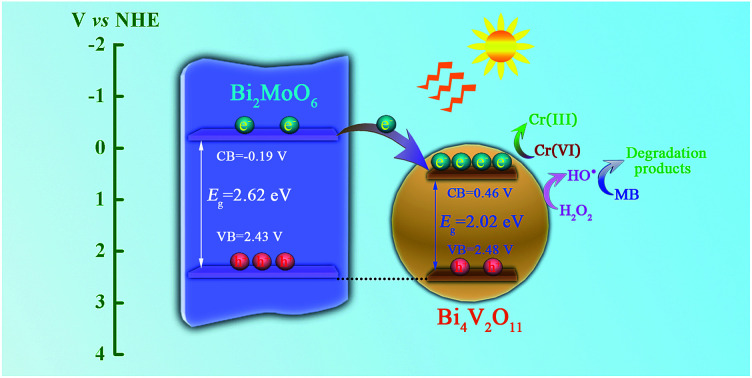

## 1. Introduction

In recent years, water pollution has become a serious problem all around the world. A large number of pollutants, such as organic dyes, heavy metal ions, drugs *etc.* are discharged into both industrial waste water and domestic sewage. Until now, the conventional water treatment methods such as adsorption, coagulation, microbial degradation, and ultra-filtration were commonly used to treat waste water, however, this approach has the disadvantage of low removal efficiency and difficulty in removing low concentrations of contaminants.^1–3^ As a kind of green energy technology, semiconductor photocatalytic technology can remove all kinds of pollutants under the irradiation of sunlight and thus, has attracted a large amount of attention. Photocatalysis possesses a number of advantages, such as the room-temperature oxidation of contaminants even at low concentrations, reduced secondary pollution, non-toxicity and low-cost, which is suitable for the degradation of contaminants.^4,5^ To date, titanium dioxide (TiO_2_) is undoubtedly considered to be the most exceptional photocatalyst for solar energy conversion and environmental applications under UV illumination (*λ* < 400 nm). As is well known, among the solar spectrum, ultraviolet-light makes up only less than 5%, while visible-light (760 nm> *λ* > 400 nm) makes up approximately 40%. However, the major drawback of TiO_2_ is the wide band gap (3.2 eV), which greatly reduces the efficiency of solar energy utilization and limits its commercial applications.^6,7^ Therefore, over the past few decades, a great deal of effort has been made to exploit more efficient visible-light-responding photocatalysts in which Bi-based semiconductors have attracted a substantial amount of attention due to their peculiar electronic structure and the low cost of raw materials.^8^

Recently, as an example of a Bi-based semiconductor, Bi_2_MoO_6_ has been regarded as a promising photocatalyst because of its narrow band gap (2.5–2.8 eV), high chemical stability and non-toxicity. Unfortunately, the rapid recombination of photo-induced electron–hole pairs and sluggish charge transport in pristine Bi_2_MoO_6_ greatly restrict its photocatalytic activity.^9,10^ To overcome the obstacles of rapid charge carrier recombination, many strategies have been developed, such as controlling the morphology,^11^ doping^12^ and constructing a heterojunction. Among them, the method of constructing a heterojunction has been the most popular, since it can boost charge carrier separation and transfer efficiently originating from the as-introduced built-in electric field at the heterostructured interface. To date, different types of Bi_2_MoO_6_-based composite photocatalysts, such as g-C_3_N_4_/Bi_2_MoO_6_,^13^ BiOCl/Bi_2_MoO_6_,^14^ BiOBr/Bi_2_MoO_6_,^15^ BiOI/Bi_2_MoO_6_,^16^ Bi_2_MoO_6_/TiO_2_,^17^ α-Fe_2_O_3_/Bi_2_MoO_6_,^18^ Bi_2_O_3_/Bi_2_MoO_6_,^19^ Bi_2_S_3_/Bi_2_MoO_6_,^20^ CdS/Bi_2_MoO_6_ (ref. 21) Bi_2_MoO_6_/Bi_2_WO_6_ (ref. 22) *etc.* have been fabricated with enhanced photocatalytic efficiency.

Very recently, Bi_4_V_2_O_11_ with a peculiar layered structure that has intrinsic oxygen vacancies in perovskite (VO_3.5_□_0.5_)^2−^ (□ represents the intrinsic oxygen vacancies) slabs sandwiched between (Bi_2_O_2_)^2+^ layers, has been considered as an excellent photocatalyst for oxygen evolution and water purification.^23–25^ Because of the narrow band gap (∼2.1 eV) and excellent charge carrier transport originating from its unique crystalline structure, Bi_4_V_2_O_11_ has been employed to construct heterojunction photocatalysts, such as BiVO_4_/Bi_4_V_2_O_11_ (ref. 26) and Bi_24_O_31_Br_10_/Bi_4_V_2_O_11_.^27^ All of above heterojunctions realize considerably enhanced photocatalytic activity due to the effective separation and transfer of photo-induced charge carriers. However, to the best of our knowledge, the construction of a Bi_2_MoO_6_/Bi_4_V_2_O_11_ heterojunction has not been reported, which might enhance the photocatalytic properties of Bi_2_MoO_6_ considerably.

In this study, a Bi_2_MoO_6_/Bi_4_V_2_O_11_ heterojunction was fabricated using a one-pot solvothermal method. This heterojunction was constructed from Bi_4_V_2_O_11_ nanocrystals anchored on the Bi_2_MoO_6_ nanoflakes surface, achieving a nanosized interfacial contact. The photocatalytic performance of the as-fabricated pristine Bi_2_MoO_6_, Bi_4_V_2_O_11_ and different Bi_2_MoO_6_/Bi_4_V_2_O_11_ heterojunctions was evaluated by the photocatalytic degradation of methylene blue (MB) and photocatalytic reduction of Cr(vi) under visible-light illumination. The results indicated that the Bi_2_MoO_6_/Bi_4_V_2_O_11_ heterojunction exhibited superior photocatalytic activity than pristine Bi_2_MoO_6_ and Bi_4_V_2_O_11_. Furthermore, the as-fabricated Bi_2_MoO_6_/Bi_4_V_2_O_11_ heterojunction exhibited good cycling stability for MB degradation after 4 cycles. Finally, the enhanced photocatalytic mechanism of the Bi_2_MoO_6_/Bi_4_V_2_O_11_ heterojunction has also been discussed.

## 2. Experimental

### 2.1 Photocatalysts preparation

All chemicals reagents were received from Aladdin Chemical Co. Ltd. and used without further purification.

2 mmol of Bi(NO_3_)_3_·5H_2_O was added into 15 mL of ethylene glycol and magnetically stirred in a water bath at 80 °C to form a clear solution. Then, appropriate stoichiometric amounts of (NH_4_)_6_Mo_7_O_24_·4H_2_O and NH_4_VO_3_ were added into the abovementioned bismuth nitrate solution, which was continuously stirred for 20 min at 80 °C to obtain a transparent solution. Subsequently, the pH of the above solution was adjusted to about 8 by the slow dropwise addition of 2 M sodium hydroxide solution. Then, the abovementioned solution was poured into a 25 mL Teflon-lined stainless autoclave, which was subsequently sealed and maintained at 160 °C for 16 h in an oven. After the reactor cooled down to room temperature, the as-obtained product was centrifuged three times using water and absolute ethanol.

Finally, the photocatalyst was obtained after drying at 80 °C in air for 4 h. According to the abovementioned method, Bi_2_MoO_6_/Bi_4_V_2_O_11_ heterojunction photocatalysts with different molar amounts of Mo and V at 0.2 mmol and 0.8 mmol (denoted as BMV-28), 0.4 mmol and 0.6 mmol (denoted as BMV-46), 0.5 mmol and 0.5 mmol (denoted as BMV-55), and 0.6 mmol and 0.4 mmol (denoted as BMV-64), respectively, were prepared. In addition, pristine Bi_2_MoO_6_ and Bi_4_V_2_O_11_ were also fabricated for the purpose of comparison.

### 2.2 Characterization

The crystalline structures of the as-fabricated samples were analyzed using X-ray diffraction (XRD) on a Rigaku D/max-2000 diffractometer with Cu K*α* radiation (*λ* = 1.5406 Å) in the range of 2*ϑ* = 20–90° at a scanning rate of 4 °C min^−1^ with a scan width of 0.02°. The morphology of the samples was observed using field emission scanning electron microscopy (FE-SEM, HELIOS NanoLab 600i) at an accelerating voltage of 20 kV. The transmission electron microscopy (TEM) and high-resolution TEM (HRTEM) analyses were carried out on a JEM-2100 transmission electron microscope at an accelerating voltage of 200 kV. The X-ray photoelectron spectroscopy (XPS) was conducted on a Thermo Scientific ESCALAB 250Xi X-ray photoelectron spectrometer coupled with a pass energy of 20.00 eV and an Al Kα excitation source (1486.6 eV). The UV-vis diffuse reflectance spectra (DRS) were obtained on a spectrophotometer (HITACHI UH-4150) using BaSO_4_ as the reflectance standard. Photoluminescence (PL) analysis was accomplished on a HORIBA FluoroMax-4.

### 2.3 Photocatalytic activity and photoelectrochemical measurements

The photocatalytic performance of the as-fabricated samples was evaluated by the degradation of methylene blue (MB) and reduction of Cr(vi) under visible-light illumination using a 300 W Xe lamp (Trusttech PLS-SXE 300, Beijing) with a UV cut-off filter (*λ* ≥ 400 nm). In a typical photocatalytic process, 0.05 g of the as-fabricated sample was added into 100 mL of MB solution (10 mg L^−1^) or Cr(vi) solution (10 mg L^−1^, which was based on Cr in a dilute K_2_Cr_2_O_7_ solution). The photocatalyst was dispersed in the above solution under ultrasonic treatment for 10 min, and the mixed solution was magnetically stirred in the dark for 30 min to achieve an adsorption–desorption equilibrium between the photocatalyst and the pollutants. When the photodegradation experiment began, 4 mL of the solution was collected from the suspension at fixed-time intervals, centrifuged at 10^4^ rpm for 5 min to remove catalyst powders, and the MB and Cr(vi) concentrations were determined using a HITACHI UH-5300 UV-vis spectrometer at 664 and 352 nm, respectively. The photoelectrochemical measurements were carried out on a CHI604C electrochemical workstation using a standard three-compartment cell with the Bi_4_V_2_O_11_, Bi_2_MoO_6_ and Bi_2_MoO_6_/Bi_4_V_2_O_11_ samples coated on FTO glass used as the working electrode, a piece of Pt sheet as the counter electrode, standard Ag/AgCl in saturated KCl as the reference electrode, and a 0.5 M Na_2_SO_4_ aqueous solution as the electrolyte. Converting between the measured potential (*vs.* Ag/AgCl) and NHE was achieved using eqn (1).^28^

1 *E*_NHE_ = *E*_Ag/AgCl_ + 0.1976 (25 °C)

The light source employed was a 300 W Xe lamp. The Mott–schottky measurements were carried out at a frequency of 100 Hz and an amplitude of 10 mV.

## 3. Results and discussion

**Fig. 1:**
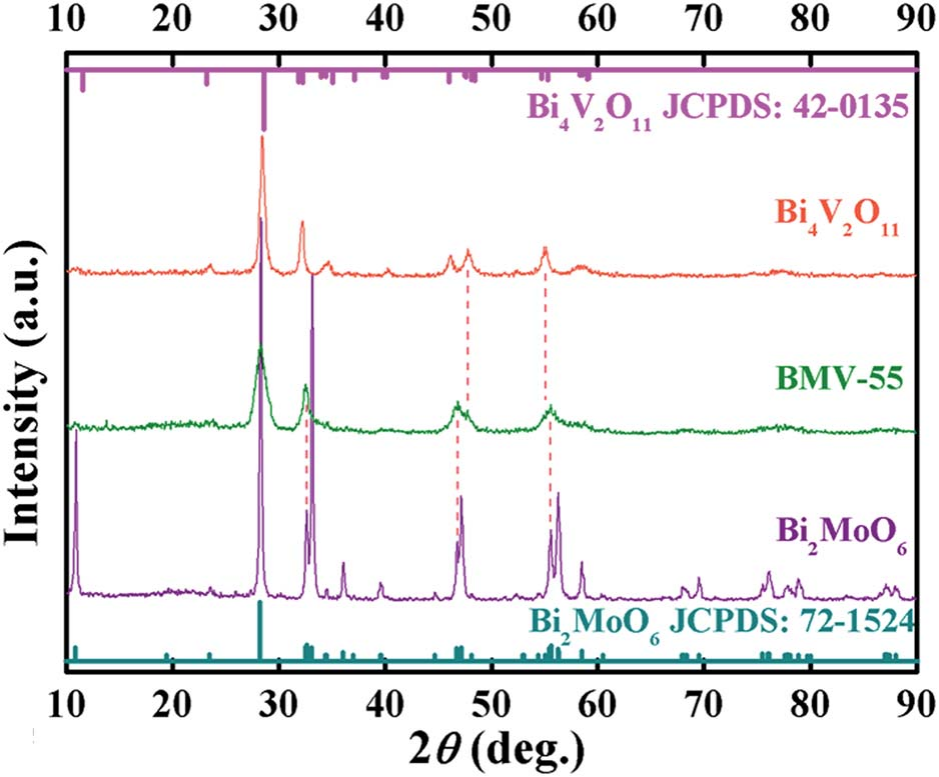
The XRD patterns of pristine Bi_2_MoO_6_ and Bi_4_V_2_O_11_ samples and the BMV-55 composite.

**Fig. 2:**
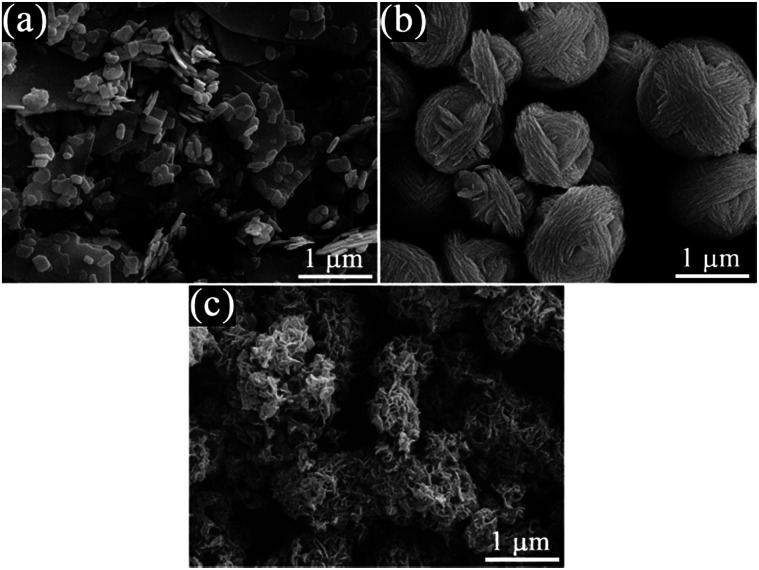
FESEM images of (a) pristine Bi_2_MoO_6_, (b) pristine Bi_4_V_2_O_11_ and (c) the BMV-55 composite.

**Fig. 3:**
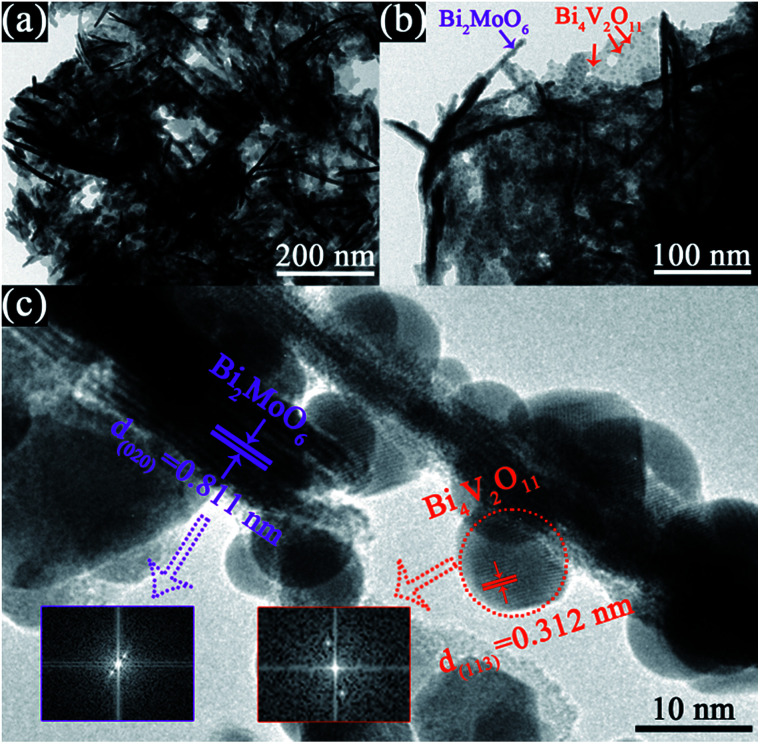
TEM images of the BMV-55 composite. The (a) TEM image at low magnification, (b) magnified TEM image, and (c) HRTEM image of the BMV-55 composite.

**Fig. 4:**
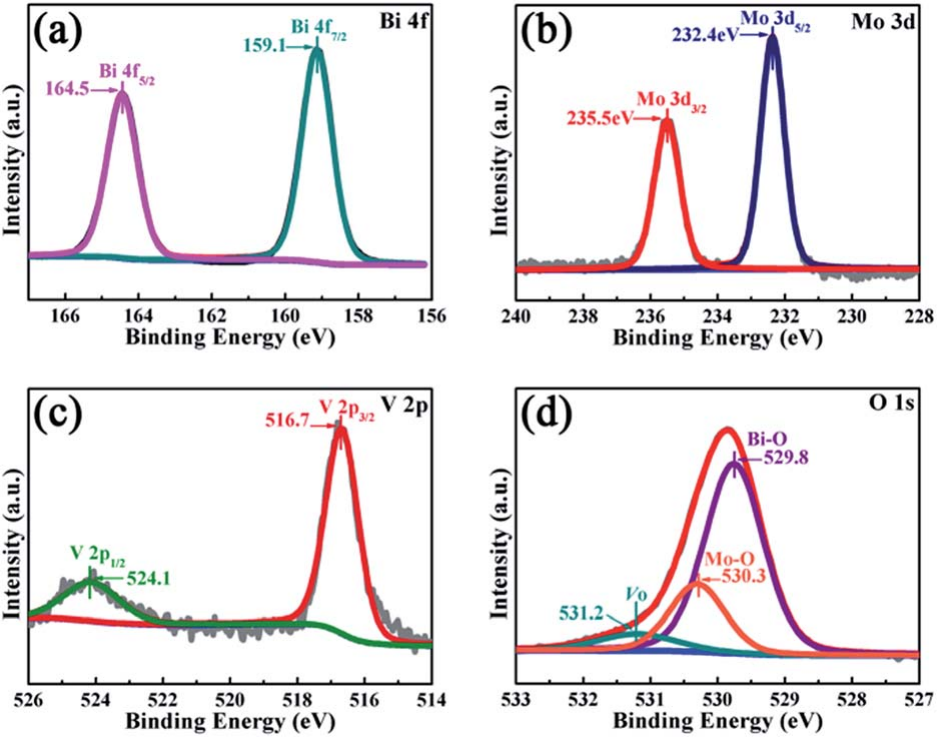
The XPS spectra of the BMV-55 composite: (a) Bi 4f, (b) Mo 3d, (c) V 2p, and (d) O 1s.

Fig. 1 shows the XRD patterns of the as-prepared pristine Bi_2_MoO_6_ and Bi_4_V_2_O_11_ samples, and the BMV-55 composite sample. The diffraction peaks of the pristine Bi_2_MoO_6_ and Bi_4_V_2_O_11_ samples can be well indexed to the orthorhombic phase Bi_2_MoO_6_ (JCPDS No. 72-1524) and the orthorhombic phase Bi_4_V_2_O_11_ (JCPDS No. 42-0135), respectively. The XRD pattern of the BMV-55 composite contains both Bi_2_MoO_6_ and Bi_4_V_2_O_11_ phases, indicating the coexistence of the two phases in the BMV-55 composite in which the extremely weak diffraction intensity of Bi_4_V_2_O_11_ can be ascribed to its tiny size and uniform dispersion on the surface of Bi_2_MoO_6_. In addition, the XRD patterns of the BMV-28, BMV-46 and BMV-64 samples are shown in Fig. S1 in the ESI.† It can be seen that the diffraction peaks of BMV-28 and BMV-46 samples belong to the characteristic peaks of Bi_4_V_2_O_11_, while no peaks corresponding to Bi_2_MoO_6_ can be detected, which might be attributed to the relatively lower content of Bi_2_MoO_6_ in the samples. Meanwhile, the diffraction peaks of BMV-64 samples correspond to those of Bi_4_V_2_O_11_; however, the peak intensities are significantly weakened relative to those of pristine Bi_4_V_2_O_11_. The morphology and microstructure of the samples are investigated using SEM, TEM and HRTEM. Fig. 2 shows the SEM images of the as-fabricated samples. The FESEM image in Fig. 2a shows that pristine Bi_2_MoO_6_ possesses an irregular flake-like morphology with a thickness of *ca.* 55 nm. In contrast, pristine Bi_4_V_2_O_11_ (Fig. 2b) displays hierarchical microspheres with diameters of approximately 1–2 μm. In addition, Fig. 2c shows the morphology of the BMV-55 composite, showing that the BMV-55 composite has a fluffy structure stacked in a disorderly manner by the nanoflakes. Moreover, the SEM images of the BMV-28, BMV-46 and BMV-64 samples are shown in Fig. S2 in the ESI.† To obtain a more detailed structural information about the BMV-55 composite, TEM analysis has been carried out. The TEM images of the BMV-55 composite are shown in Fig. 3. The TEM image of the BMV-55 composite at low magnification (Fig. 3a) illustrates that the BMV-55 composite is comprised of nanoflakes with a thickness of about 10 nm and length of about 100–200 nm, which is consistent with the SEM results described above. Interestingly, as observed from the magnified TEM image (Fig. 3b), nanocrystals with a size of *ca.* 10 nm are homogeneously dispersed and tightly adhered to the surface of the nanoflakes. By observing the HRTEM image of the BMV-55 composite (Fig. 3c), it can be further confirmed that the nanoflakes are Bi_2_MoO_6_ and the as-anchored nanocrystals are Bi_4_V_2_O_11_ because the observed interplanar spacings of 0.811 nm and 0.312 nm correspond well to the (020) plane of the orthorhombic Bi_2_MoO_6_ and the (113) plane of the orthorhombic Bi_4_V_2_O_11_, respectively. These results suggest that the Bi_4_V_2_O_11_ nanocrystals are embedded on the Bi_2_MoO_6_ nanoflakes in the BMV-55 composite, forming a Bi_2_MoO_6_/Bi_4_V_2_O_11_ heterojunction with a nanosized interfacial contact. With the aim of further verifying the coexistence of Bi_2_MoO_6_ and Bi_4_V_2_O_11_, surface electronic states analysis of the BMV-55 composite have been performed using the X-ray photoelectron spectroscopy (XPS). Fig. 4 shows the high-resolution XPS spectra of the as-fabricated BMV-55 composite photocatalyst. The full survey spectra and C 1s spectra of BMV-55 are shown in Fig. S3 in the ESI.†

**Fig. 5:**
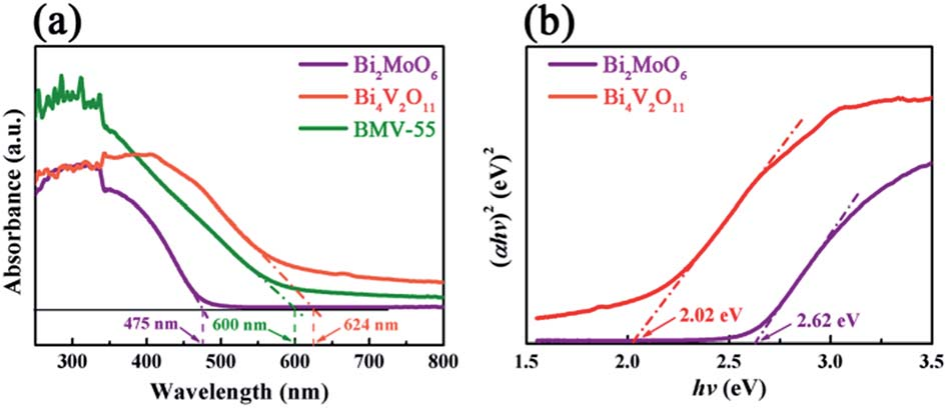
The (a) UV-vis diffuse reflectance spectra (DRS) and (b) (*αhν*)^2^*vs. hν* curves of the pristine Bi_2_MoO_6_ and Bi_4_V_2_O_11_ samples, and the BMV-55 composite.

The peak positions in all of the XPS spectra were calibrated using the C 1s peak (284.6 eV) as the reference. First, the two characteristic peaks located at 164.5 and 159.1 eV in the Bi 4f spectra (Fig. 4a) were assigned to Bi 4f_5/2_ and Bi 4f_7/2_, respectively, which corresponded to Bi^3+^.^29^ Then, in the Mo 3d XPS spectra (Fig. 4b), the binding energies for Mo 3d_3/2_ and Mo 3d_5/2_ located at 235.5 and 232.4 eV, respectively were assigned to Mo^6+^ in Bi_2_MoO_6_.^9,29^ Furthermore, for the V 2p peaks (Fig. 4c), the two peaks located at 524.1 and 516.7 eV were assigned to V 2p_1/2_ and V 2p_3/2_, respectively and belonged to V^5+^ in Bi_4_V_2_O_11_.^27,30^ Moreover, as shown in Fig. 4d, the O 1s spectra could be divided into three peaks, and the binding energies located at 529.8, 530.3 and 531.2 eV could be assigned to the lattice oxygen in Bi–O,^13^ lattice oxygen in Mo–O,^18^ and intrinsic oxygen vacancy (*V*_o_) in Bi_4_V_2_O_11_,^31^ respectively. The XPS results further indicated the simultaneous existence of Bi_2_MoO_6_ and Bi_4_V_2_O_11_ species in the BMV-55 composite photocatalyst. In a bid to investigate the optical absorption capacity of the as-prepared photocatalysts, UV-vis diffuse reflection spectra (DRS) analysis was carried out and is displayed in Fig. 5. As can be seen from Fig. 5a, the absorption edges of pristine Bi_2_MoO_6_, Bi_4_V_2_O_11_ and the BMV-55 composite were observed at about 475, 624 and 600 nm, respectively. Obviously, the absorption edge of the BMV-55 composite exhibited a red shift compared with that of pristine Bi_2_MoO_6_, which illustrated that the introduction of Bi_4_V_2_O_11_ was conducive to enhancing the light absorption capacity of the BMV-55 composite. In addition, the band gaps of pristine Bi_2_MoO_6_ and Bi_4_V_2_O_11_ were calculated using the Kubelka–Munk function (eqn (2)).^32^

**Fig. 6:**
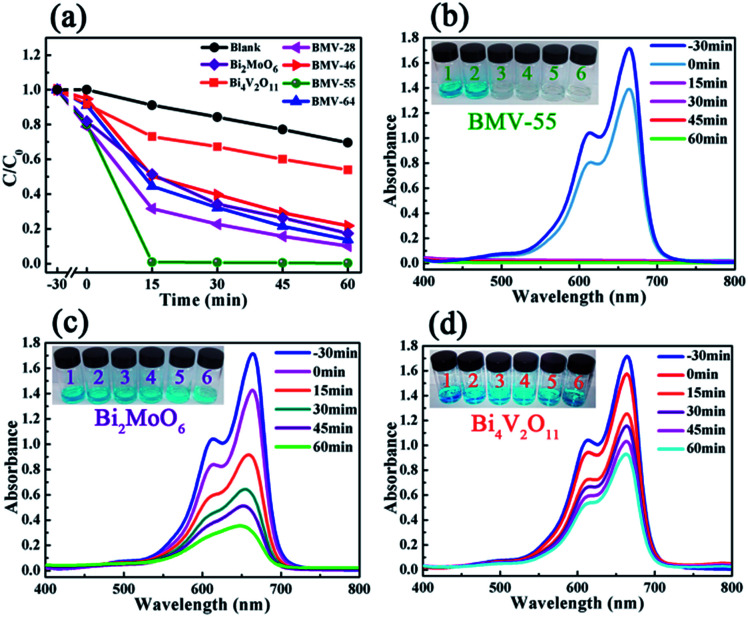
(a) The photocatalytic degradation plots of MB over the various photocatalysts in the presence of H_2_O_2_. The UV-vis absorption spectral changes observed during the degradation of MB over (b) BMV-55, (c) pristine Bi_2_MoO_6_ and (d) pristine Bi_4_V_2_O_11_.

2 *αhν* = *A*(*hν* − *E*_g_)^*n*/2^

where *α*, *h*, *ν*, *A*, and *E*_g_ represent the absorption coefficient, Planck constant, light frequency, a constant, and band gap, respectively. In this function, *n* is determined by the type of optical transition in the semiconductor. Generally, *n* = 1 for direct transition and *n* = 4 for indirect transition. Both Bi_2_MoO_6_ (ref. 21) and Bi_4_V_2_O_11_ (ref. 33) are direct transition semiconductors and their *n* value is 1. Fig. 5b shows the curves of (*αhν*)^2^*vs.* photon energy (*hν*) of the pristine Bi_2_MoO_6_ and Bi_4_V_2_O_11_ samples, which indicate that the band gaps of pristine Bi_2_MoO_6_ and Bi_4_V_2_O_11_ are approximately 2.62 and 2.02 eV, respectively. The photocatalytic performance of the as-prepared photocatalysts was evaluated by the degradation of MB in the presence of H_2_O_2_ (0.1 mL) under visible light irradiation. Fig. 6a presents the variation plots of MB concentration (*C*/*C*_0_) *versus* light irradiation time for the different Bi_2_MoO_6_/Bi_4_V_2_O_11_ composites along with the pristine Bi_2_MoO_6_ and Bi_4_V_2_O_11_ samples. As shown in the figure, the degradation rate of MB over the pristine Bi_2_MoO_6_ and Bi_4_V_2_O_11_ samples reached *ca.* 83% and 46% after 60 min, respectively. Among the as-fabricated Bi_2_MoO_6_/Bi_4_V_2_O_11_ composites, with the exception of BMV-55, there was no obvious enhancement in the photocatalytic efficiency over the three other samples (BMV-28, BMV-46 and BMV-64) compared with that of pristine Bi_2_MoO_6_. In contrast, the BMV-55 composite exhibited a significantly enhanced photocatalytic activity towards the degradation of MB among the samples and the photodegradation rate of MB achieved was almost 100% after 15 min of light irradiation. Fig. 6b–d display the UV-vis absorption spectra of the degradation solution obtained at certain time intervals over the BMV-55 composite and pristine Bi_2_MoO_6_ and Bi_4_V_2_O_11_ samples, respectively, which showed the superiority of the BMV-55 composite for MB degradation relative to those of pristine Bi_2_MoO_6_ and Bi_4_V_2_O_11_ samples. To explore the effect of H_2_O_2_ on the degradation efficiency of MB, the degradation experiment of MB without the addition of H_2_O_2_ was also conducted, and the result is shown in Fig. S4.†

**Fig. 7:**
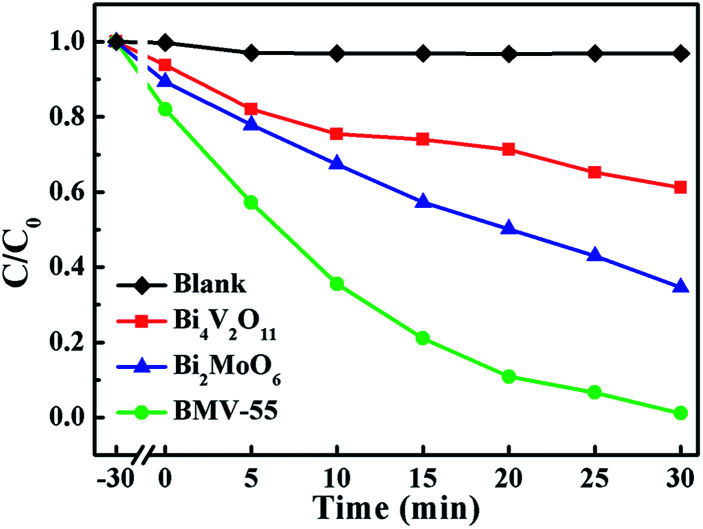
The photocatalytic Cr(vi) reduction plots over pristine Bi_2_MoO_6_ and Bi_4_V_2_O_11_, and the BMV-55 composite samples under visible-light irradiation.

**Fig. 8:**
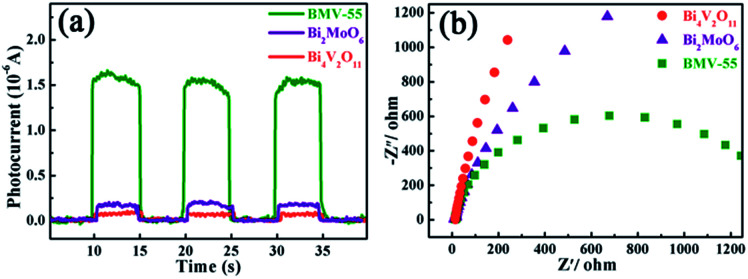
The (a) transient photocurrent plots and (b) electrochemical impedance spectroscopy (EIS) Nyquist plots obtained for the pristine Bi_2_MoO_6_ and Bi_4_V_2_O_11_, and the BMV-55 composite samples.

**Fig. 9:**
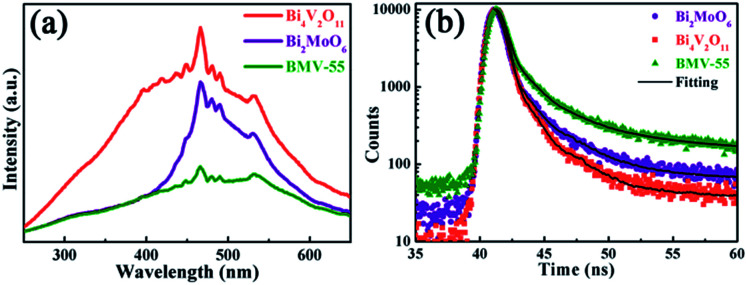
(a)The PL spectra of pristine Bi_2_MoO_6_ and Bi_4_V_2_O_11_, and the BMV-55 composite (*λ*_ex_ = 345 nm). (b) The time-resolved fluorescence decay spectra and corresponding fitting curves of pristine Bi_2_MoO_6_ and Bi_4_V_2_O_11_, and the BMV-55 composite.

Obviously, the photocatalytic activity of BMV-55 in the absence of H_2_O_2_ is much lower than that found upon adding H_2_O_2_ into the system of which the degradation rate is only 75% after 90 min, indicating that H_2_O_2_ plays a key role in our photocatalytic reactions. To further study the photocatalytic application of the as-fabricated photocatalysts, we have also used a toxic heavy metal ion, Cr(vi), as a model pollutant, and we have conducted the photocatalytic reduction of Cr(vi) over the pristine Bi_2_MoO_6_ and Bi_4_V_2_O_11_ samples, and the BMV-55 composite samples with the results illustrated in Fig. 7. As can be seen from the figure, the BMV-55 composite shows a distinctly enhanced photocatalytic activity for the reduction of Cr(vi) compared with pristine Bi_2_MoO_6_ and Bi_4_V_2_O_11_, demonstrating that our heterojunction photocatalysts have a good application in wastewater purification. It is speculated that the highest activity of the BMV-55 composite could be associated with the higher separation and transport efficiency of the photo-induced carriers originating from the construction of the heterojunction between the Bi_2_MoO_6_ nanoflakes and Bi_4_V_2_O_11_ nanocrystals. Aiming at confirming this viewpoint, two important approaches, including photoelectrochemical and photoluminescence (PL) analyses, have been used to evaluate the separation and transport efficiency of photo-induced carriers. Fig. 8 illustrates the results of the electrochemical measurements consisting of the photocurrent and electrochemical impedance spectroscopy (EIS) over the as-prepared pristine Bi_2_MoO_6_ and Bi_4_V_2_O_11_ samples, and the BMV-55 composite, under visible irradiation. As demonstrated in Fig. 8a, the BMV-55 composite exhibits a remarkably enhanced photocurrent response in striking contrast with the pristine Bi_2_MoO_6_ and Bi_4_V_2_O_11_ samples. In detail, the photocurrent intensity of the BMV-55 composite is approximately 8- and 18-fold larger than that of pristine Bi_2_MoO_6_ and Bi_4_V_2_O_11_, implying that the separation and transport of the photoexcited charge carriers can be effectively promoted by the construction of the heterogeneous structure by anchoring the Bi_4_V_2_O_11_ nanocrystals on the Bi_2_MoO_6_ nanoflakes. Fig. 8b shows the EIS Nyquist plots. Apparently, the BMV-55 composite exhibits a smaller arc radius relative to that of the pristine Bi_2_MoO_6_ and Bi_4_V_2_O_11_ samples, indicating the significantly enhanced interfacial charge carrier transport ability of the BMV-55 composite. These results can furnish the valid evidence that the BMV-55 composite possesses facilitated charge carrier separation and transport properties compared with pristine Bi_2_MoO_6_ and Bi_4_V_2_O_11_. To shed light on the recombination rate and fluorescence lifetime of the photo-induced charge carriers, PL analysis has been performed, as shown in Fig. 9. As depicted in Fig. 9a, the BMV-55 composite exhibits a lower PL emission intensity compared with pristine Bi_2_MoO_6_ and Bi_4_V_2_O_11_ samples, manifesting that the recombination of the photo-induced charge carriers is effectively suppressed in the Bi_2_MoO_6_/Bi_4_V_2_O_11_ heterojunction. Moreover, Fig. 9b presents the time-resolved fluorescence decay spectra monitored at 460 nm under an excitation wavelength of 345 nm and the corresponding fitting curve of pristine Bi_2_MoO_6_ and Bi_4_V_2_O_11_, and the BMV-55 composite. Obviously, the decay kinetics of the BMV-55 composite are slower when compared to those of pristine Bi_2_MoO_6_ and Bi_4_V_2_O_11_. Consequently, the radiative lifetimes are extracted by means of a reconvolution fit using a 3-exponential model (eqn (3)).^34^3
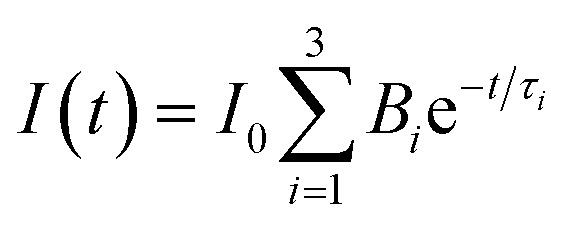
where *t* is the decay time after the absorption, *I* (*t*) is the fluorescence intensity at time *t*, *I*_0_ is the intensity at time *t* = 0, *τ*_*i*_ is the lifetime and *B*_*i*_ is the pre-exponential factor. In addition, the averaged-lifetime *τ̄* is calculated using eqn (4).^34^4
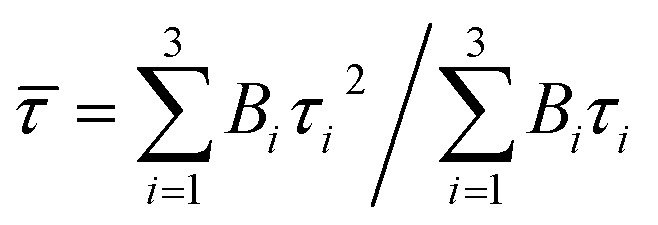


The best fitting curve and kinetic parameters observed for pristine Bi_2_MoO_6_ and Bi_4_V_2_O_11_, and the BMV-55 composite are displayed in Fig. S5 in the ESI.† In detail, the average-lifetime of carriers in pristine Bi_2_MoO_6_ and Bi_4_V_2_O_11_ were shortened to *ca.* 1.983 and 0.487 ns, respectively. In contrast, the average-lifetime of the BMV-55 composite was distinctly prolonged to 7.059 ns, indicating that the formation of the Bi_2_MoO_6_/Bi_4_V_2_O_11_ heterojunction is tremendously beneficial to prolong the radiative lifetimes of the photo-induced charge carriers, and the greatly prolonged lifetime of the charge carriers could be closely related to the high separation efficiency of the charge carriers. The results of the PL spectra and time-resolved fluorescence decay spectra analysis further reveal that the BMV-55 composite exhibits a significantly enhanced separation and transport efficiency for the photo-induced charge carriers relative to those of pristine Bi_2_MoO_6_ and Bi_4_V_2_O_11_, which is very consistent with the results of the photoelectrochemical analysis.

**Fig. 10:**
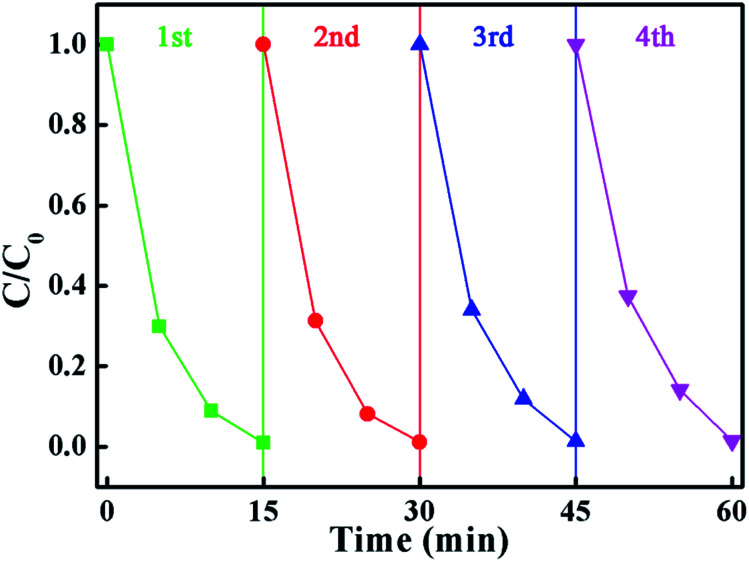
The results of the recycling experiment after 4 cycles for the photodegradation of MB in the presence of H_2_O_2_ over the BMV-55 composite sample.

From a practical point of view, the cycle stability of the photocatalysts is a very important factor in evaluating the photocatalytic performance. To evaluate the cycle stability, a cycling experiment for the photodegradation of MB over the BMV-55 composite upon adding 0.1 mL of H_2_O_2_ was carried out. Prior to the next cycle experiment, the previously used photocatalyst was subjected to ultrasonic cleaning with distilled water and drying at 60 °C. Fig. 10 depicts the results of the recycling experiment after 4 cycles. It was be clearly seen that the photodegradation rate of MB for 15 minutes remained above 95% after 4 cycles, which indicated that the as-fabricated BMV-55 composite photocatalyst possessed good cycle stability. For the purpose of unraveling the mechanism of the enhanced photocatalytic activity of the Bi_2_MoO_6_/Bi_4_V_2_O_11_ heterojunction, the energy band structures of pristine Bi_2_MoO_6_ and Bi_4_V_2_O_11_ were investigated by determining their band gaps and flat-band potentials. Using the above UV-vis diffuse reflection spectra (DRS) analysis (Fig. 5b), the band gaps of pristine Bi_2_MoO_6_ and Bi_4_V_2_O_11_ were determined to be 2.62 and 2.02 eV, respectively.

**Fig. 11:**
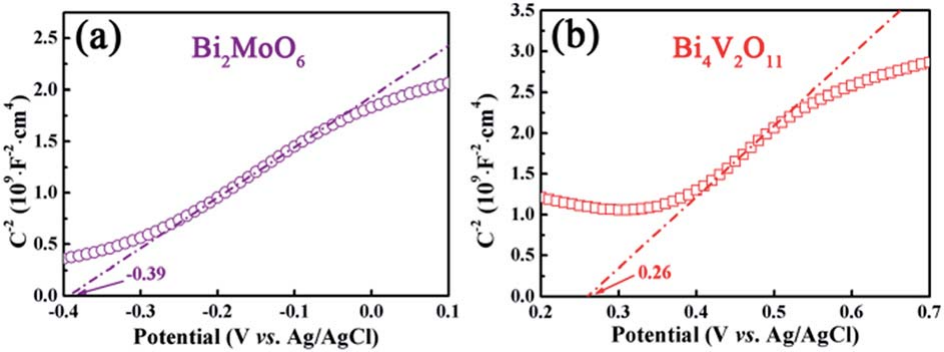
The Mott–Schottky plots of (a) pristine Bi_2_MoO_6_ and (b) pristine Bi_4_V_2_O_11_.

**Scheme 1:**
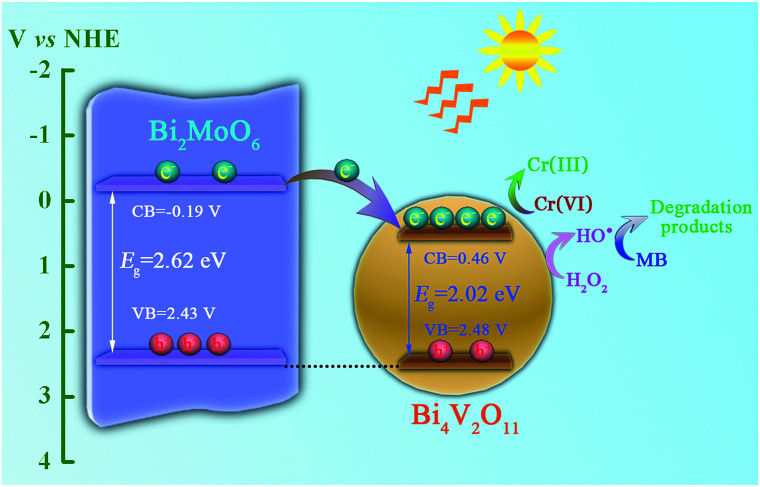
A schematic illustration of the photo-induced charge carrier separation and photocatalytic reaction mechanism over the Bi_2_MoO_6_/Bi_4_V_2_O_11_ heterojunction.

In addition, the flat-band potentials of pristine Bi_2_MoO_6_ and Bi_4_V_2_O_11_ are determined *via* Mott–Schottky analysis, and the results are shown in Fig. 11. It can be seen that both of them show a positive slop, indicating that they are all n-type semiconductors. In addition, the flat-band potentials of pristine Bi_2_MoO_6_ and Bi_4_V_2_O_11_ are *ca.* −0.39 and 0.26 V, respectively, *vs.* Ag/AgCl (−0.19 and 0.46 V *vs.* NHE), which indicates that the flat-band potential of Bi_2_MoO_6_ is more negative than that of Bi_4_V_2_O_11_ by 0.65 V. According to the semiconductor theory, the conduction band potential (*E*_CB_) of n-type semiconductors is very close to (0.1–0.2 V more negative) their flat-band potential.^35^ Therefore, it can be concluded that the CB position of Bi_2_MoO_6_ is more negative than that of Bi_4_V_2_O_11_. Based on above results, possible photo-induced electron–hole pair separation and the photocatalytic degradation of MB mechanism over the Bi_2_MoO_6_/Bi_4_V_2_O_11_ heterojunction with the assistance of H_2_O_2_ as an electron-trapping agent are proposed, as shown in Scheme 1. On one hand, because of the fact that the CB potential of Bi_2_MoO_6_ is more negative than that of Bi_4_V_2_O_11_, the photo-induced electrons can easily migrate from the CB of Bi_2_MoO_6_ to the CB of Bi_4_V_2_O_11_ driven by the built-in electric field at the heterostructured interface; it could be then captured by H_2_O_2_ and reacted to generate hydroxide radicals (˙OH) with strong oxidizing ability, which will completely degrade MB. On the other hand, since the VB potentials of Bi_2_MoO_6_ (2.43 V) and Bi_4_V_2_O_11_ (2.48 V) are very close to each other, the migration of the photo-induced holes is very difficult, leading to the holes staying in their valence band positions.^36^ In this way, the rapid migration of the photo-induced electrons and the immobilization of the photo-induced holes can lead to the effective separation of the photo-induced electrons and holes over the Bi_2_MoO_6_/Bi_4_V_2_O_11_ heterojunction, which ultimately results in the enhanced photocatalytic activity observed during the photodegradation of MB.

## 4. Conclusions

In summary, a novel visible-light responsive Bi_2_MoO_6_/Bi_4_V_2_O_11_ heterojunction photocatalyst with a nanosized interfacial contact has been successfully fabricated using a facile one-pot solvothermal method. The Bi_2_MoO_6_/Bi_4_V_2_O_11_ heterojunctions show excellent photocatalytic MB degradation efficiency under visible-light illumination. In particular, the BMV-55 composite exhibits significantly enhanced photocatalytic activity during the photodegradation of MB and photoreduction of Cr(vi) compared with pristine Bi_2_MoO_6_ and Bi_4_V_2_O_11_. In addition, the Bi_2_MO_6_/Bi_4_V_2_O_11_ heterojunctions also display an enhanced transient photocurrent response, lower electrochemical impedance, lower PL intensity and greatly prolonged lifetime, which indicates that the photo-induced charge carriers are more effectively separated and transferred in the Bi_2_MoO_6_/Bi_4_V_2_O_11_ heterojunctions. The significantly enhanced photocatalytic activity and charge carrier separation effect are attributed to the formation of a heterojunction with a nanosized interfacial contact between the Bi_2_MoO_6_ nanoflakes and Bi_4_V_2_O_11_ nanocrystals. Moreover, the Bi_2_MoO_6_/Bi_4_V_2_O_11_ heterojunctions exhibit excellent cycle stability after 4 cycles. This work may be further extended to the research and development of a novel semiconductor heterojunction containing a new kind of Bi_4_V_2_O_11_ photocatalyst for water purification and related applications.

## Conflicts of interest

There are no conflicts to declare.

## Supplementary Material

RA-008-C7RA12766A-s001
