# Supplementary material for: The synthesis of a Bi2MoO6/Bi4V2O11 heterojunction photocatalyst with enhanced visible-light-driven photocatalytic activity
Source: RSC Adv. 2018 Jan 31;8(10):5433–40. doi: 10.1039/c7ra12766a (PMC9078112; doi:10.1039/c7ra12766a)
Supplement: RA-008-C7RA12766A-s001 [file RA-008-C7RA12766A-s001.pdf]

Electronic Supplementary Material (ESI) for RSC Advances.
This journal is © The Royal Society of Chemistry 2018

Supporting Information

Synthesis of $Bi_2MoO_6/Bi_4V_2O_{11}$ heterojunction photocatalyst with enhanced visible-light-driven photocatalytic activity

Chol-Nam Ri*, Song-Gol Kim, Kyong-Sik Ju, Hyok-Su Ryo, Chol-Ho Mun, U-Hyon Kim

Institute for electronic materials, Kim Il Sung University, Pyongyang, 999093, Democratic People's Republic of Korea

*Corresponding Author: rcn906@yahoo.com

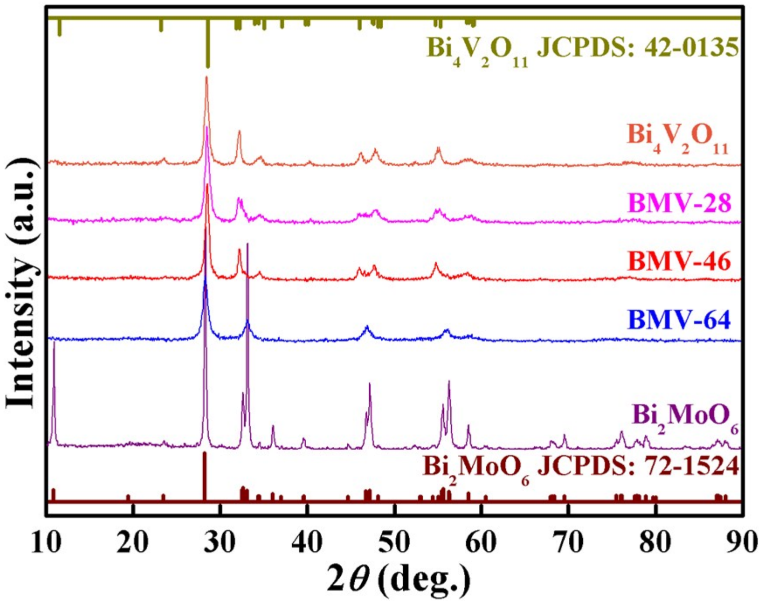

Fig. S1. XRD patterns of pristine Bi_2MoO_6, $Bi_4V_2O_{11}$ and BMV-28, BMV-46, BMV-64 composite samples.

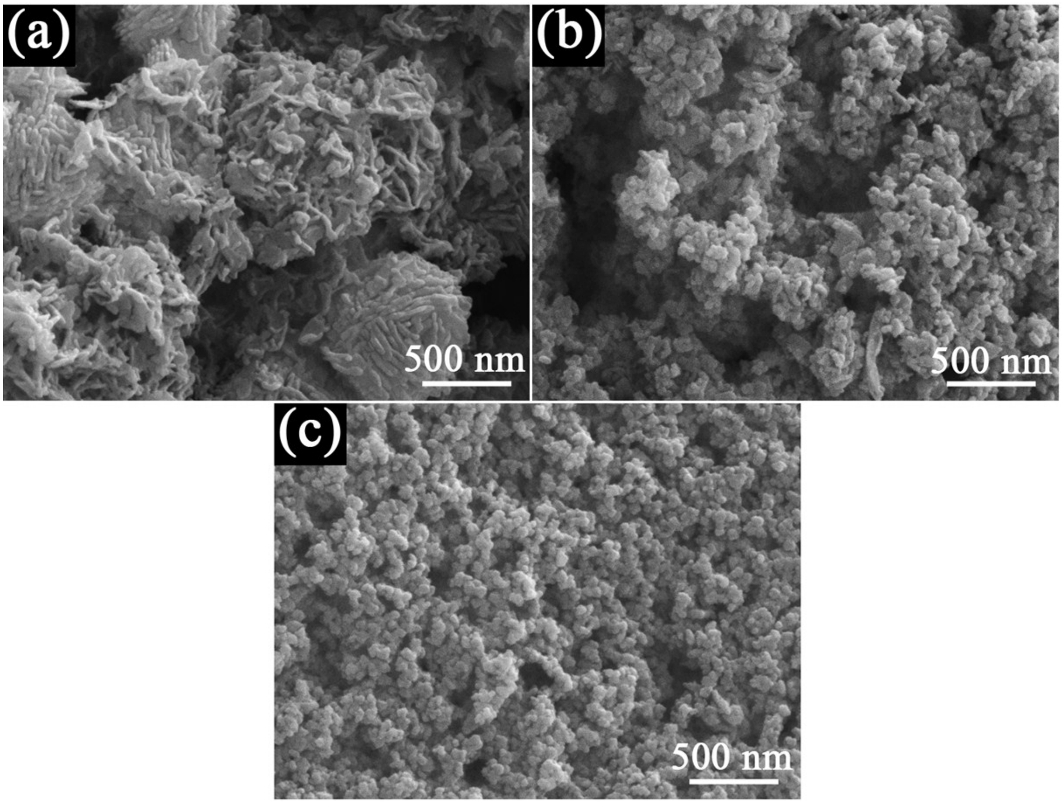

Fig. S2. SEM images of (a) BMV-28, (b) BMV-46 and BMV-64 composite samples.

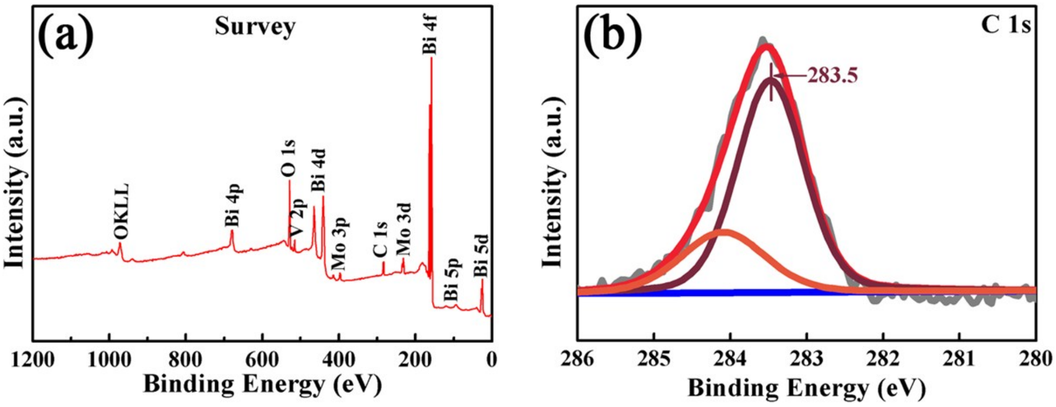

Fig. S3. (a) Full survey spectra and (b) C ls spectra of BMV-55 composite.

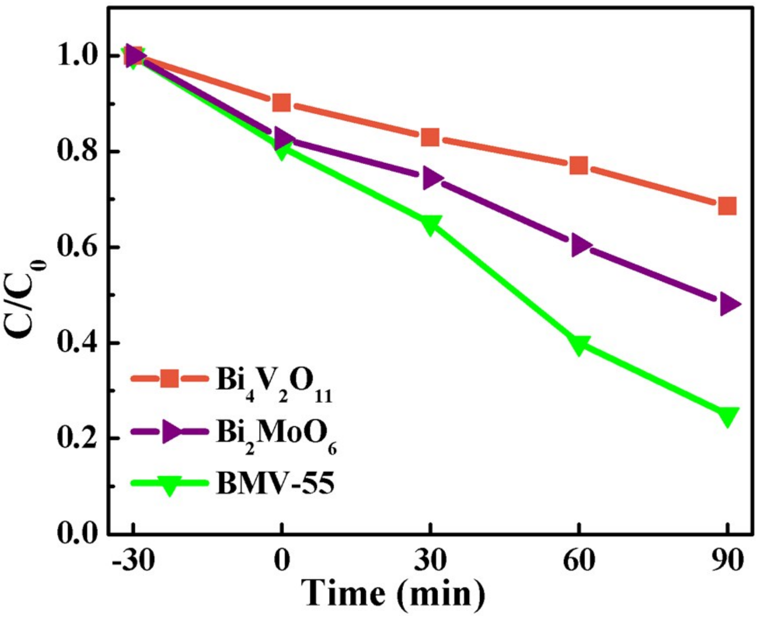

Fig. S4. Photocatalytic degradation plots of MB in the absence of H_2O_2 over pristine Bi_2MoO_6, Bi_4V_2O11, BMV-55 composite samples.

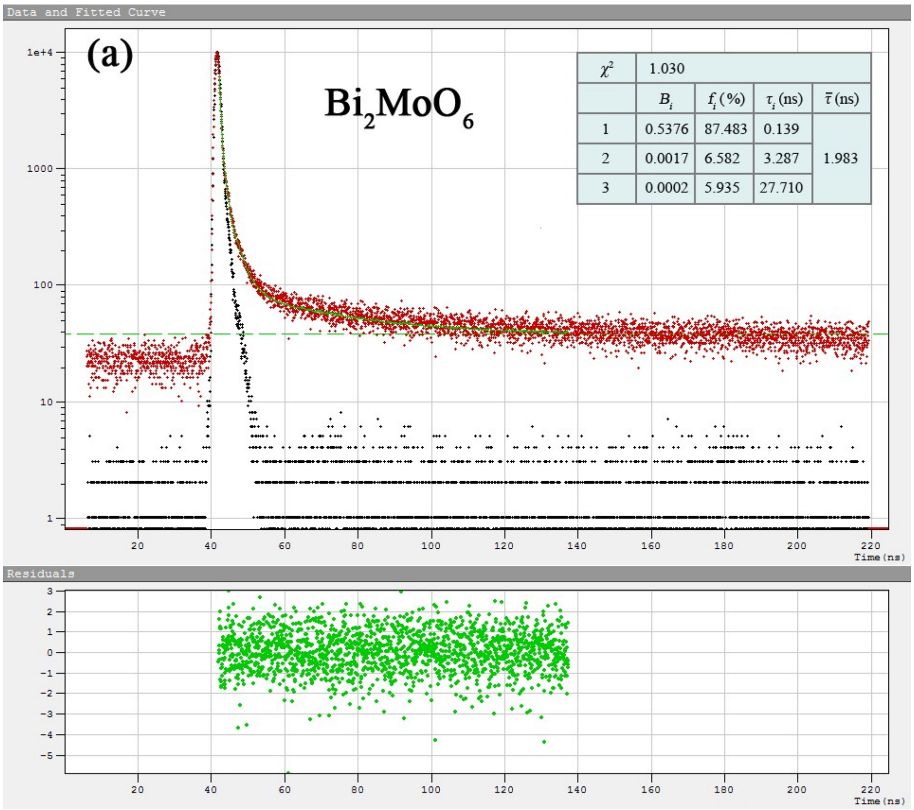

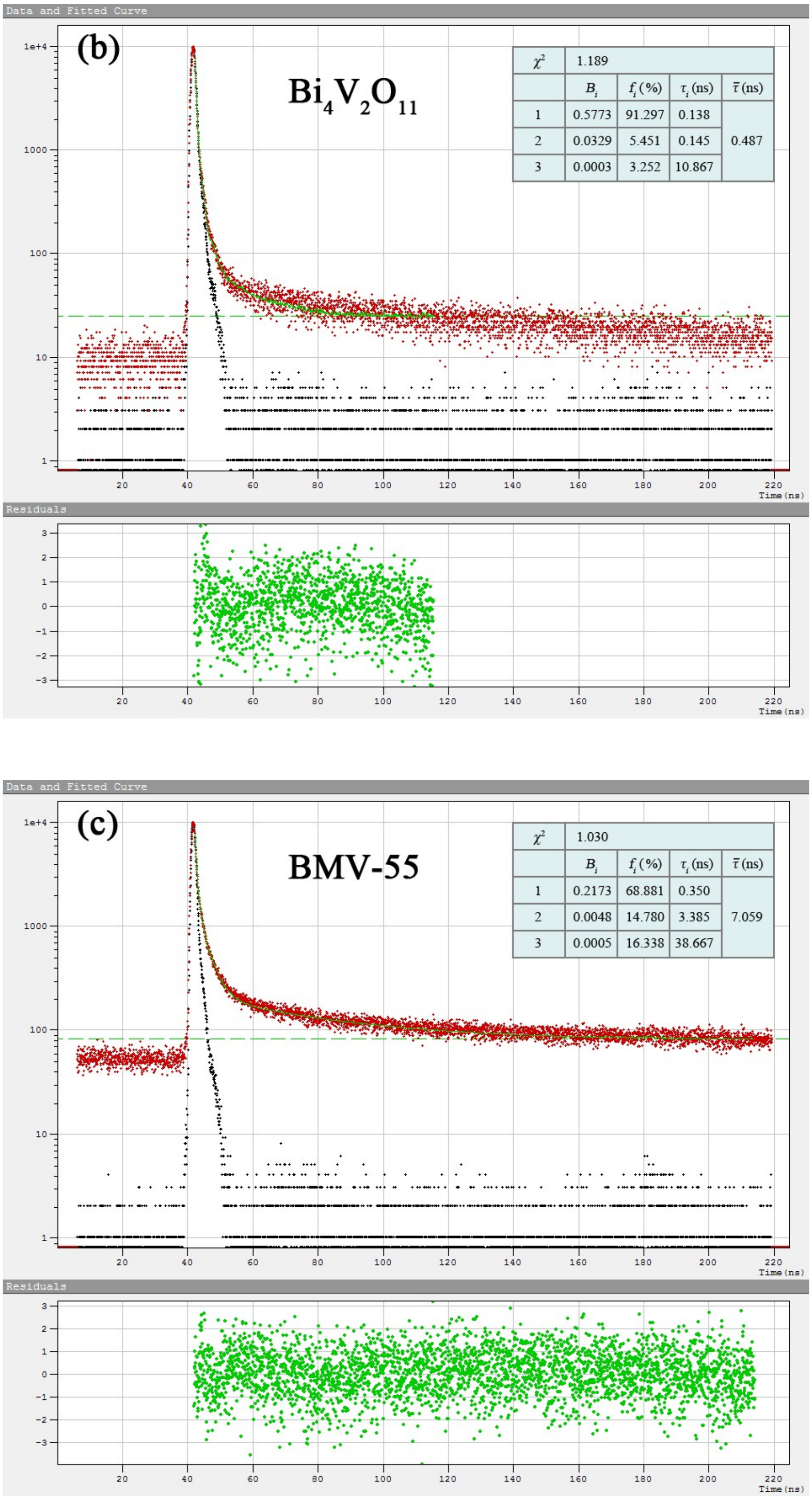

Fig. S5. Fitting curves using 3-exponential model and resulting of kinetic parameters; (a) as-fabricated pristine Bi_2MoO_6, (b) $Bi_4V_2O_{11}$ and (c) BMV-55 composite samples.
